# Animal food intake and cooking methods in relation to endometrial cancer risk in Shanghai

**DOI:** 10.1038/sj.bjc.6603458

**Published:** 2006-10-24

**Authors:** W-H Xu, Q Dai, Y-B Xiang, G-M Zhao, W Zheng, Y-T Gao, Z-X Ruan, J-R Cheng, X-O Shu

**Affiliations:** 1 Department of Epidemiology, Shanghai Cancer Institute, 2200/25 Xie Tu Road, Shanghai 200032, China; 2 Department of Medicine, Vanderbilt Epidemiology Center, Vanderbilt University, Nashville TN 37232-8300, USA; 3 Department of Epidemiology, Fudan University School of Public Health, Shanghai 200032, China

**Keywords:** endometrial cancer, dietary factor, case–control study

## Abstract

We evaluated animal food intake and cooking methods in relation to endometrial cancer risk in a population-based case–control study in Shanghai, China. A validated food frequency questionnaire was used to collect the usual dietary habits of 1204 cases and 1212 controls aged 30–69 years between 1997 and 2003. Statistical analyses were based on an unconditional logistic regression model adjusting for potential confounders. High intake of meat and fish was associated with an increased risk of endometrial cancer, with adjusted odds ratios for the highest *vs* the lowest quartile groups being 1.7 (95% confidence interval: 1.3–2.2) and 2.4 (1.8–3.1), respectively. The elevated risk was observed for all types of meat and fish intake. Intake of eggs and milk was not related to risk. Cooking methods and doneness levels for meat and fish were not associated with risk, nor did they modify the association with meat and fish consumption. Our study suggests that animal food consumption may play an important role in the aetiology of endometrial cancer, but cooking methods have minimal influence on risk among Chinese women.

Endometrial cancer, a hormone-dependent disease, has an incidence rate in developing countries, such as China, more than 10 times lower than that in developed countries (Parkin *et al*, 2003). However, established hormonal risk factors, including menopausal status, parity, oral contraceptive use, hormone replacement therapy and body mass index, can only explain part of the large international variation in its incidence (Kaaks *et al*, 2002). Ecological studies suggest that dietary habits have a major contribution to this international variation (Armstrong and Doll, 1975; Parkin, 1989). Several studies found that high consumption of red meat was associated with increased risk of endometrial cancer (Levi *et al*, 1993; Shu *et al*, 1993; Goodman *et al*, 1997), whereas others found no association (La Vecchia *et al*, 1986; Zheng *et al*, 1995; McCann *et al*, 2000; Jain *et al*, 2000). Three studies reported that high dietary intake of fish was inversely associated with risk (La Vecchia *et al*, 1986; McCann *et al*, 2000; Terry and Wolk *et al*, 2002), two found no association (Levi *et al*, 1993; Goodman *et al*, 1997), and two others observed a positive association (Shu *et al*, 1993; Zheng *et al*, 1995). Intake of eggs was related to an elevated risk in some (Levi *et al*, 1993, Zheng *et al*, 1995; Goodman *et al*, 1997; Terry and Vainio *et al*, 2002) but not in other studies (La Vecchia *et al*, 1986). On the other hand, consumption of milk was not associated with any increased risk in most studies (La Vecchia *et al*, 1986; Zheng *et al*, 1995; Goodman *et al*, 1997; Jain *et al*, 2000; McCann *et al*, 2000; Terry and Vainio *et al*, 2002) but was related to reduced risk in two studies (Barbone *et al*, 1993; Petridou *et al*, 2002). Most studies, however, have been conducted in Western countries, and none have evaluated potential interactions with cooking methods, which may partly explain the inconsistency in previous studies. *In vivo* and *in vitro* studies have suggested that meats cooked at a high temperature generate heterocyclic amines (HCAs) and other potent mutagens and carcinogens, which can induce tumours in multiple tissue sites in many animal species (Spingarn *et al*, 1980; Wakabayashi *et al*, 1992; Okochi *et al*, 1999; Nagao *et al*, 2002). Although a number of epidemiologic studies suggest a possible link between dietary intake of HCAs and risk of colorectal and breast cancers (Zheng *et al*, 1998; Nowell *et al*, 2002; Sinha *et al*, 2005), no studies have been conducted on endometrial cancer.

In a large case–control study, we investigated whether dietary intake of animal foods is associated with endometrial cancer risk and whether this is modified by cooking methods.

## SUBJECTS AND METHODS

Eligible cases for the study were residents of urban Shanghai, aged 30–69 years, newly diagnosed with endometrial cancer between 1997 and 2003. A total of 1454 endometrial cancer cases were identified through the Shanghai Cancer Registry, and 1204 of them were interviewed, yielding a response rate of 82.8%. All cases were confirmed either by histopathology or by medical history review. Excluded from the analysis were 135 cases who refused to be interviewed (9.3%), 66 who died before interview (4.5%), 23 (1.6%) who could not be located, 12 who were out of town during the period of interview (0.8%), and 14 who could not be interviewed for other miscellaneous reasons (1.0%). The median interval between diagnosis and interview was 5.6 months.

Controls were randomly selected from the Shanghai Resident Registry and frequency matched to cases according to age distribution of endometrial cancer cases in 1996. After exclusion of 63 women who reported a hysterectomy, 1212 out of 1629 eligible controls (74.4%) consented and were interviewed for the study. Reasons for non-participation among controls were refusal (*n*=340, 20.9%), being out of town during the period of interview (*n*=61, 3.7%), severe illness (*n*=13, 0.8%), and other miscellaneous reasons (*n*=3, 0.2%).

Study participants were interviewed in person by trained nurses and physicians, using a structured questionnaire to obtain information on demographic characteristics, menstrual and reproductive events, exogenous hormone use, diet, cigarette smoking, alcohol consumption, physical activity, family history of cancer, and medical history. Direct body measurements on body height, weight, and waist and hip size were conducted at the time of interview. The quantitative food frequency questionnaire (FFQ) used in the study has been validated by two FFQ surveys and one 24-h diet recall survey (Shu *et al*, 2004). The FFQ listed 76 food items commonly consumed in Shanghai, including 19 animal food items which were classified into the following 10 food groups: red meat, organ meat, poultry, marine fish, fresh water fish, shrimp and crab, eel, shellfish, eggs, and milk. Red meat included pork, beef, and mutton meat. Each subject was asked to report her usual intake frequency (per day, week, month, or year) and amount in liangs (50 g) for each food item over the past 5 years, ignoring any recent changes. The participant was also asked whether she used deep-frying, stir-frying or roasting/grilling methods to prepare meats and fish, in addition to how frequently she used each cooking method in cooking these foods. The subject was also asked to report whether she usually deep-fried meat and fish to the level that (1) the surface still had a bloody colour; (2) virtually no surface was brown; (3) a small portion of the surface was brown (Goodman *et al*, 1997); the majority of the surface was brown; or (5) the entire surface was brown with a slightly burnt flavour. Levels 4 and 5 were referred to as well-done.

Consumption of common animal foods was categorised into four groups based on the quartile distribution among controls. Uncommon animal foods, such as organ meat, eel, shellfish, and milk were grouped into two levels, never and ever. The Wilcoxon rank sum test was used for comparisons of the median difference between cases and controls and the *χ*^2^-test was used for categorical variables. An unconditional logistic regression model was applied to obtain maximum likelihood estimates of the odds ratios (ORs) and their 95% confidence intervals (CIs). All of the ORs were adjusted for age (as a continuous variable), menopausal status (pre/post menopausal), diagnosis of diabetes (ever/never), alcohol consumption (ever/never), body mass index (BMI) (as a continuous variable), physical activity in metabolic equivalent tasks (as a continuous variable), and total energy intake (as a continuous variable). Other variables, including dietary intake of total vegetables and soy foods, did not appreciably alter the risk estimates and were not adjusted for in our analyses. Tests for trend across quartiles were performed in a logistic regression model by assigning a numerical score (1–4) to each category of categorised variables. Stratified analyses were conducted to evaluate potential modifying effects.

## RESULTS

Table 1 shows comparisons of cases and controls for demographic factors and known risk factors of endometrial cancer. No significant differences were observed between cases and controls for age, income, marital status, use of hormone replacement therapy, mean intake of vegetables, or intake of fruits and soy protein. Compared with controls, cases were more likely to have attended higher education, have an earlier age at menarche, a later age at menopause, more years of menstruation, and to be pre-menopausal. Cases were also more likely to be diagnosed with diabetes, have a family history of cancer, have a higher total energy intake and a higher BMI, and were less likely to have had a pregnancy, engaged in regular exercise, used oral contraceptives, or consumed alcohol regularly (Table 1).

Presented in Table 2 are comparisons of the average intake levels of total and individual animal foods between cases and controls. The median intakes of red meat, poultry, marine fish, fresh water fish, shrimp and crab, eel, and shellfish were significantly higher in cases than in controls. No significant differences were found for intake of organ meat, eggs, and milk.

The associations between animal food intake and endometrial cancer risk are presented in Table 3. The ORs were 1.7 (95% CI: 1.3–2.2) and 2.4 (95% CI: 1.8–3.1), respectively, for those who ate total meat and total fish in the highest intake quartile as compared with those in the lowest quartile. A positive association was observed for consumption of red meat, poultry, marine fish, fresh water fish, shrimp and crab. Consumption of organ meat, eel, shellfish, and milk was not very common in our study population. A moderately increased risk (OR=1.3, 95% CI: 1.1–1.6) was found to be associated with eel or shellfish consumption. Egg and milk consumption were not associated with endometrial cancer risk. No case–control difference was found regarding ever/never eating organ meat. The consumption of organ meat in this study population was too low to evaluate disease risk by intake level.

Neither cooking method nor doneness level of meat and fish were associated with endometrial cancer risk, nor did they modify the association of meat and fish intake with disease risk (Table 4). Additional analyses stratified by consumption of total vegetables and soyfood did not show substantial changes in the above-mentioned associations (data not shown).

## DISCUSSION

The positive association of disease with intake of red meat and the null association with eggs and milk found in the current study are consistent with those found in previous studies (La Vecchia *et al*, 1986; Levi *et al*, 1993; Shu *et al*, 1993; Zheng *et al*, 1995; Goodman *et al*, 1997; McCann *et al*, 2000; Jain *et al*, 2000; Terry and Vainio *et al*, 2002). However, our finding of a positive association with intake of poultry was inconsistent with previous reports in which null (Okochi *et al*, 1999; McCann *et al*, 2000) or inverse (Goodman *et al*, 1997) associations were reported. Our observation of an elevated risk associated with a high consumption of fish, although inconsistent with some previous studies (La Vecchia *et al*, 1986; Levi *et al*, 1993; Goodman *et al*, 1997; McCann *et al*, 2000; Terry and Wolk *et al*, 2002), is in line with that from our earlier report on the same disease (Shu *et al*, 1993). Endometrial cancer is a hormone-dependent disease. It has been suggested that dietary fat is responsible for the association between animal foods and endometrial cancer risk (Levi *et al*, 1993; Goodman *et al*, 1997), probably through influencing oestrogen metabolism and enhancing oestrogen re-absorption in the bowel (Gorbach and Goldin, 1987), although the relation between dietary fat and endogenous oestrogen levels remains controversial (Holmes *et al*, 2000). *In vitro* and *in vivo* studies have found that omega-3 fatty acids, which fish, particularly marine fish, are rich in, inhibit the proliferation and progression of hormone-related cancer (Ip, 1997; Rose and Connolly, 1999). On the other hand, omega-6 fatty acids were found to increase synthesis of cyclooxygenase- and lipoxygenase-catalysed products and thus may increase cancer risk (Nair *et al*, 1997). It has been shown that farm-raised fish are high in omega-6 but low in omega-3 fatty acids (van Vliet and Katan, 1990). The high consumption of fresh water fish in our study population compared to the high intake of marine fish among Western populations may explain the contradictory findings on fish consumption in our study compared to those conducted in Western countries.

Chemical pollution may be another explanation for our finding on fish. Fish growing in polluted water may have a high level of methylmercury, polychlorinated dibenzo-*p*-dioxins, dibenzofurans, organochlorine residues, and other chemicals. Some of these chemicals have high toxicity and carcinogenic potency (Rojas *et al*, 1999). Fresh-water fish have been found to be contaminated with methyl mercury, organochlorines, and other chemicals in China (Hou *et al*, 1988; Nakata *et al*, 2002). Evidence that organochlorines are present in seafood is also available (Jiang *et al*, 2005). Several studies conducted in Shanghai, including this one, have consistently found that fish intake is related to an increased risk of several cancers, including cancers of breast, endometrium, and colorectum (Shu *et al*, 1993; Guo *et al*, 1994; Dai *et al*, 2002; Chiu *et al*, 2003), suggesting that water pollution may be involved in the aetiology of these cancers. Organochlorines have been shown to have estrogenic activity (Olea *et al*, 1998; Brouwer *et al*, 1999) and may increase the risk of hormone-related cancers such as prostate and breast cancer (Terry *et al*, 2003). However, results from two studies that have directly evaluated the association between organochlorines and endometrial cancer found either no significant association (Weiderpass *et al*, 2000) or a negative association (Sturgeon *et al*, 1998). Further studies are needed to search for the factors that are responsible for the positive association between fish consumption and cancer risk in this population.

Meat and fish cooked at high temperatures can produce HCAs, potent experimental mutagens, or carcinogens (Dolara *et al*, 1979; Sinha and Knize *et al*, 1998; Sinha and Rothman *et al*, 1998; Wong *et al*, 2005). Heterocyclic amines begin to form at temperatures of 150°C or higher, and their production can be increased up to three-fold when the cooking temperature is increased from 200 to 250°C (Dolara *et al*, 1979). Frying, broiling, grilling, and baking are associated with formation of large amounts of HCAs (Sinha and Knize *et al*, 1998; Sinha and Rothman *et al*, 1998). In our population, deep-frying is the most common high-temperature method for cooking red meat and fish, whereas roasting or grilling is less common, although it may be used to cook poultry. Oil temperature is normally around 240–270°C during deep-frying. It has been reported that deep-frying also generates fumes containing mutagenic compounds (de Meester and Gerber, 1995; Shields *et al*, 1995). These mutagens or carcinogens have been linked to an increased risk of cancers among Western populations (Zimmerli *et al*, 2001; Sinha *et al*, 2005). However, in our study, we found no association between cooking patterns or doneness levels and the risk of endometrial cancer, nor did they modify the risk associated with meat intake with the exception of the positive association with deep-fried cooking observed among women whose consumption of fish was in the lowest quartile. The latter is likely to be attributed to a chance finding owing to the lack of an underlying biological mechanism and the multiple comparisons conducted in this study. Our findings may reflect the fact that deep-frying, roasting, and grilling are much less common among the Chinese compared to people living in Western countries (Koh *et al*, 2005). In Shanghai, stir-frying is the most common cooking practice. Thus, the exposure to HCAs in our study population may not have been high enough to increase cancer risk.

Compared with Western populations, our study population has a much lower level of meat consumption. On average, the total meat intake is only about half to one-third of that consumed in Western countries (Sinha *et al*, 2005; Gonzalez *et al*, 2006). Our findings provide potent evidence of the unfavourable role of meat intake in the development of endometrial cancer, even at a low average level of consumption, and thus strongly support public health messages suggesting that women should be encouraged to lower their meat intake.

The strengths of our study include a population-based design, a large sample size, high response rates, and the availability of detailed dietary information collected using a validated FFQ. Given the nature of the case–control study design, however, recall bias is a major concern in our study. In this study, the response rate among controls (74.4%) is lower than that in cases (82.8%), raising a concern about selective participation bias. Although neither the potential study participants nor the interviewers knew our study hypotheses, we cannot exclude the possibility that study participation would be influenced by general health knowledge including healthy dietary habits. However, we found that the controls of the current study had patterns of meat and fish intake similar to the controls of the Shanghai Breast Cancer Study which was conducted in the same population 2–3 years before the current study which had a much higher response rate (91.1% for cases and 90.3% for controls) (Dai *et al*, 2002). Therefore, selection bias is unlikely to be the main explanation for the study findings. The dietary intake of cases may have changed as a result of cancer diagnosis and treatment, and current diet may influence the recall of usual diet (Willett, 1998). In China, cancer patients are commonly advised to avoid or lower meat intake for improving prognosis. Thus, dietary change after cancer diagnosis would probably lead to an underestimation of the association of meat intake with endometrial cancer risk. In our study, the median interval between diagnosis and interview for cases was only 5.6 months. Study participants were asked to report their dietary habits before cancer diagnosis, ignoring any recent dietary changes. This may have minimised the misclassification of the dietary assessment. However, we cannot exclude the possibility that some patients might link meat intake to the cause of their disease and thus the result may have been over-reporting of meat consumption among cases.

In summary, this large population-based case–control study of endometrial cancer, conducted in a population that has a much lower total meat consumption compared to people in Western countries, found that diets high in animal foods increase the risk of endometrial cancer.

## Figures and Tables

**Table 1:** Comparison between cases and controls of demographics and selected risk factors in Shanghai Endometrial Cancer Study

	**Cases (*n*=1204)**	**Controls (*n*=1212)**	***P*-value**
Age (Mean±s.d.)^a^	54.5±8.5	54.6±8.5	0.77
			
*Education levels (%)* b			
No formal education	95 (8.0)	133 (11.0)	
Primary school	170 (14.1)	157 (12.9)	
Middle school	446 (37.0)	441 (36.4)	
High school	311 (25.8)	326 (27.0)	
College or above	182 (15.1)	155 (12.8)	0.05
			
*Per capita income in previous year (yuan) (%)* b			
<4166.67	321 (26.7)	353 (29.1)	
4166.68–6250.00	317 (26.3)	317 (26.2)	
6250.01–8750.00	261 (21.7)	267 (22.0)	
>8750.00	305 (25.3)	275 (22.7)	0.11
			
*Marital status (%)* b			
Unmarried	18 (1.5)	13 (1.1)	
Married or cohabiting	1055 (87.6)	1062 (87.6)	
Separated/divorced/widowed	131 (10.9)	137 (11.3)	0.63
			
*Menopausal status (%)* b			
No	502 (41.7)	447 (36.9)	
Yes	702 (58.3)	765 (63.1)	0.02
			
Age at menarche (mean±s.d.)^a^	14.5±1.7	14.8±1.8	<0.01
Age at menopause (mean±s.d.)^a^	50.2±3.6	49.0±3.8	<0.01
Years of menstruation (mean±s.d.)^a^	32.8±4.9	30.6±5.4	<0.01
Number of pregnancies (mean±s.d.)^a^	2.6±1.5	2.9±1.5	<0.01
			
*Regularly exercise (%)* b			
Never	859 (71.4)	806 (66.5)	
Ever	345 (28.6)	406 (33.5)	0.01
			
*Oral contraceptive use (%)* b			
Never	981 (81.5)	910 (75.1)	
Ever	223 (18.5)	302 (24.9)	<0.01
			
*Any cancer history among the first-degree relatives (%)* b			
No	774 (64.8)	867 (72.1)	
Yes	420 (35.2)	336 (27.9)	<0.01
			
*Cigarette smoking (%)* b			
Never	1163 (96.6)	1170 (96.5)	
Ever	41 (3.4)	42 (3.5)	0.94
			
*Alcohol consumption* (*%)*^b^			
Never	1170 (97.2)	1147 (94.6)	
Ever	34 (2.8)	65 (5.4)	<0.01
			
Body height (m) (mean±s.d.)^a^	1.58 (0.06)	1.57 (0.05)	<0.01
Body weight (kg) (mean±s.d.)^a^	64.0 (10.7)	58.8 (9.0)	<0.01
BMI (kg m^2^) (mean±s.d.)^a^	25.7±4.1	23.8±3.5	<0.01
			
*Hormone replacement therapy (%)* b			
Never	1151 (95.6)	1162 (96.0)	
Ever	53 (4.4)	49 (4.0)	0.66
			
*Diabetes mellitus* b			
Never	1009 (84.7)	1121 (93.1)	
Ever	182 (15.3)	83 (6.9)	<0.01
			
Total vegetable intake (mean±s.d.)^a^	328.1±205.8	325.4±206.2	0.75
Total fruit intake (mean±s.d.)^a^	225.5±174.1	223.0 ±195.4	0.75
Soy protein intake (mean±s.d.)^a^	11.7±8.4	12.0±9.1	0.46
Total energy intake (mean±s.d.)^a^	1086.5±466.7	1763.5±468.8	0.02

BMI=body mass index; s.d.=standard deviation.

Subjects with missing values were excluded from the analysis.

a *P*-value for *t*-test.

b *P*-value for *χ*^2^-test.

**Table 2:** Intake levels of animal foods among cases and controls in Shanghai Endometrial Cancer Study

	**Median (25th, 75th percentile)**		
**Animal foods**	**Cases (*n*=1204)**	**Controls (*n*=1212)**	***P*-value^a^**
Total meat	59.7 (37.0, 89.3)	52.1 (31.2, 81.7)	<0.01
Red meat	43.7 (25.7, 67.1)	38.6 (22.4, 61.9)	<0.01
Organ meat	0.1 (0.0, 1.3)	0.0 (0.0, 1.2)	0.25
Poultry	10.9 (4.6, 22.0)	8.7 (4.0, 18.5)	<0.01
			
Fish, shrimp, crab, and eel	47.3 (24.6, 78.9)	35.9 (17.6, 65.7)	<0.01
Marine fish	12.1 (4.8, 26.2)	10.5 (3.6, 26.2)	<0.01
Fresh water fish	12.6 (4.8, 21.1)	9.7 (3.1, 21.1)	<0.01
Shrimp and crab	5.8 (2.9, 16.7)	4.8 (1.9, 12.5)	<0.01
Eel	0.5 (0.0, 3.5)	0.2 (0.0, 2.3)	<0.01
			
Shellfish	0.3 (0.0, 1.1)	0.2 (0.0, 1.1)	0.02
Eggs	25.0 (12.5, 43.7)	25.0 (12.5, 43.7)	0.56
Milk	9.9 (0.0, 25.0)	10.7 (0.0, 25.0)	0.76

a *P*-value: Wilcoxon rank sum test.

**Table 3:** Odds ratios and 95% CIs for animal food intake in association with endometrial cancer risk

	**Quartile of intake (g day^−1^)**				
**Food groups**	**Q1 (Low)**	**Q2**	**Q3**	**Q4 (High)**	***P*-value for trend**
*Total meat intake*					
Cases/controls	224/303	271/303	348/303	361/303	
OR1	1.0	1.2 (0.9–1.5)	1.5 (1.2–1.9)	1.6 (1.3–2.0)	<0.01
OR2	1.0	1.2 (0.9–1.6)	1.6 (1.3–2.1)	1.7 (1.3–2.2)	<0.01
					
*Red meat*					
Cases/controls	238/303	290/303	325/303	351/303	
OR1	1.0	1.2 (0.9–1.5)	1.4 (1.1–1.7)	1.4 (1.1–1.8)	<0.01
OR2	1.0	1.2 (0.9–1.5)	1.4 (1.1–1.8)	1.4 (1.1–1.9)	<0.01
OR3^a^	1.0	1.2 (0.9–1.5)	1.3 (1.0–1.7)	1.3 (1.0–1.8)	0.02
					
*Organ meat*					
Cases/controls	598/625 (Never)	606/587 (Ever)			
OR1	1.0	1.1 (0.9–1.3)			
OR2	1.0	1.1 (0.9–1.4)			
OR3^a^	1.0	1.1 (0.9–1.4)			
					
*Poultry*					
Cases/controls	254/303	282/335	285/272	383/302	
OR1	1.0	1.0 (0.8–1.2)	1.3 (1.0–1.6)	1.5 (1.2–1.9)	<0.01
OR2	1.0	1.0 (0.8–1.3)	1.4 (1.1–1.8)	1.6 (1.3–2.1)	<0.01
OR3^a^	1.0	1.0 (0.8–1.3)	1.4 (1.1–1.8)	1.6 (1.2–2.0)	<0.01
					
*Total fish*					
Cases/controls	183/303	291/303	317/303	413/303	
OR1	1.0	1.7 (1.3–2.1)	1.9 (1.5–2.4)	2.3 (1.8–3.0)	<0.01
OR2	1.0	1.7 (1.3–2.2)	1.9 (1.4–2.4)	2.4 (1.8–3.1)	<0.01
					
*Marine fish*					
Cases/controls	295/341	244/269	407/391	258/211	
OR1	1.0	1.0 (0.8–1.3)	1.2 (1.0–1.5)	1.4 (1.1–1.8)	<0.01
OR2	1.0	1.0 (0.8–1.3)	1.2 (0.9–1.5)	1.3 (1.0–1.7)	0.02
OR3^b^	1.0	1.0 (0.8–1.3)	1.1 (0.9–1.4)	1.2 (0.9–1.5)	0.18
					
*Fresh water fish*					
Cases/controls	217/303	305/360	391/338	291/211	
OR1	1.0	1.2 (0.9–1.5)	1.6 (1.3–2.0)	1.9 (1.5–2.4)	<0.01
OR2	1.0	1.2 (0.9–1.5)	1.6 (1.3–2.0)	1.9 (1.5–2.5)	<0.01
OR3^b^	1.0	1.2 (0.9–1.5)	1.6 (1.3–2.0)	1.9 (1.4–2.4)	<0.01
					
*Shrimp and crab*					
Cases/controls	265/320	319/294	282/326	338/272	
OR1	1.0	1.3 (1.0–1.6)	1.0 (0.8–1.3)	1.5 (1.2–1.9)	<0.01
OR2	1.0	1.4 (1.1–1.7)	1.1 (0.9–1.4)	1.6 (1.2–2.0)	0.01
OR3^b^	1.0	1.3 (1.0–1.7)	1.0 (0.8–1.3)	1.4 (1.0–1.8)	0.12
					
*Eel*					
Cases/controls	515/594 (Never)	689/618 (Ever)			
OR1	1.0	1.3 (1.1–1.5)			
OR2	1.0	1.3 (1.1–1.6)			
OR3^b^	1.0	1.3 (1.1–1.6)			
					
*Shellfish*					
Cases/controls	470/541 (Never)	734/671 (Ever)			
OR1	1.0	1.3 (1.1–1.5)			
OR2	1.0	1.3 (1.1–1.6)			
					
Eggs					
Cases/controls	326/356	342/303	409/401	127/152	
OR1	1.0	1.2 (1.0–1.5)	1.1 (0.9–1.4)	0.9 (0.7–1.2)	0.81
OR2	1.0	1.2 (1.0–1.6)	1.1 (0.9–1.4)	0.9 (0.7–1.2)	0.81
					
*Milk*					
Cases/controls	493/466 (Never)	711/746 (Ever)			
OR1	1.0	0.9 (0.8–1.1)			
OR2	1.0	1.0 (0.8–1.2)			

BMI=body mass index; CI=confidence interval.

OR1: Adjusted for age.

OR2: Adjusted for age, menopausal status, diagnosis of diabetes, alcohol consumption, BMI, physical activity, and total energy intake.

a OR3: Additionally adjusted for meat intake other than target meat (as a continuous variable).

b OR3: Additionally adjusted for fish intake other than target fish (as a continuous variable).

**Table 4:** Joint effect of meat and fish intake (g day^−1^) with frequency of deep-frying and barbecuing and level of doneness

	**Use of deep-fried and barbecue cooking method**					
	**Ever**			**Well done**		
**Amount of consumption in tertile groups**	**Never**	**<1 per month**	**⩾1 per month**	**Never**	**< 1 per month**	**⩾1 per month**
*Red meat*						
⩽27.2	193/234	99/124	37/46	193/234	91/104	35/41
	1.0	0.9 (0.7–1.3)	1.0 (0.6–1.6)	1.0	1.0 (0.7–1.3)	1.0 (0.6–1.6)
27.3–52.1	178/189	143/117	89/99	178/189	123/95	76/93
	1.1 (0.8–1.4)	1.5 (1.1–2.1)	1.1 (0.8–1.6)	1.0 (0.8–1.3)	1.5 (1.1–2.0)	0.9 (0.7–1.4)
>52.1	175/162	129/100	160/141	175/162	111/81	140/128
	1.2 (0.9–1.7)	1.5 (1.1–2.2)	1.4 (1.0–1.9)	1.1 (0.9–1.5)	1.5 (1.0–2.1)	1.2 (0.9–1.7)
*P*-value for interaction test		0.54			0.61	
						
*Poultry*						
⩽5.4	168/222	124/143	36/44	168/222	112/124	35/43
	1.0	1.1 (0.8–1.5)	1.2 (0.7–1.9)	1.0	1.1 (0.8–1.6)	1.1 (0.7–1.9)
5.5–13.1	130/152	128/118	119/131	130/152	116/105	116/123
	1.1 (0.8–1.6)	1.6 (1.1–2.2)	1.2 (0.9–1.7)	1.1 (0.8–1.5)	1.6 (1.1–2.2)	1.2 (0.9–1.7)
>13.1	144/117	140/95	214/190	144/117	128/81	200/178
	1.7 (1.2–2.4)	2.1 (1.5–3.0)	1.5 (1.1–2.0)	1.6 (1.2–2.3)	2.2 (1.5–3.1)	1.5 (1.1–1.9)
*P*-value for interaction test		0.71			0.71	
						
*Total fish*						
⩽23.9	143/241	75/113	114/110	143/241	63/93	103/100
	1.0	1.2 (0.8–1.7)	1.7 (1.2–2.5)	1.0	1.0 (0.7–1.5)	1.4 (1.0–2.0)
24.0–53.0	104/136	81/74	131/120	104/136	69/57	114/109
	1.3 (0.9–1.8)	2.0 (1.4–3.0)	1.9 (1.4–2.7)	1.1 (0.8–1.5)	1.8 (1.2–2.7)	1.5 (1.1–2.1)
>53.0	179/139	103/86	270/193	179/139	90/71	243/170
	2.3 (1.6–3.1)	2.1 (1.5–3.1)	2.5 (1.8–3.3)	1.8 (1.4–2.5)	1.8 (1.3–2.6)	2.0 (1.5–2.7)
*P*-value for interaction test		0.29			0.32	

BMI=body mass index; OR=odds ratio.

OR: Adjusted for age, menopausal status, diagnosis of diabetes, alcohol consumption, physical activity, BMI, and total energy intake.

## References

[bib1] Armstrong B, Doll R (1975) Environmental factors and cancer incidence and mortality in different countries, with special reference to dietary practices. Int J Cancer 15: 617–631 1140864 10.1002/ijc.2910150411

[bib2] Barbone F, Austin H, Partridge EE (1993) Diet and endometrial cancer: a case–control study. Am J Epidemiol 137: 393–403 8460621 10.1093/oxfordjournals.aje.a116687

[bib3] Brouwer A, Longnecker MP, Birnbaum LS, Cogliano J, Kostyniak P, Moore J, Schantz S, Winneke G (1999) Characterization of potential endocrine-related health effects at low-dose levels of exposure to PCBs. Environ Health Perspect 107: 639–649 10421775 10.1289/ehp.99107s4639 PMC1567499

[bib4] Chiu BCH, Ji BT, Dai Q, Gridley G, Mlaughlin JK, Gao YT, Fraumeni Jr JF, Chou WH (2003) Dietary factors and risk of colon cancer in Shanghai, China. Cancer Epidemiol Biomarkers Prev 12: 201–208 12646508

[bib5] Dai Q, Shu XO, Jin F, Gao YT, Ruan ZX, Zheng W (2002) Consumption of animal foods, cooking methods, and risk of breast cancer. Cancer Epidemiol Biomarkers Prev 11: 801–808 12223422

[bib6] de Meester C, Gerber GB (1995) The role of cooked food mutagens as possible etiological agents in human cancer. A critical appraisal of recent epidemiological investigations. Rev Epidemiol Sante Publique 43: 147–161 7732201

[bib7] Dolara P, Commoner B, Vithayathil A, Cuca G, Tuley E, Madyastha P, Nair S, Kriebel D (1979) The effect of temperature on the formation of mutagens in heated beef stock and cooked ground beef. Mutat Res 60: 231–237 384210 10.1016/0027-5107(79)90013-7

[bib8] Gonzalez CA, Jakszyn P, Pera G, Agudo A, Bingham S, Palli D, Ferrari P, Boeing H, del Giudice G, Plebani M, Carneiro F, Nesi G, Berrino F, Sacerdote C, Tumino R, Panico S, Berglund G, Siman H, Nyren O, Hallmans G, Martinez C, Dorronsoro M, Barricarte A, Navarro C, Quiros JR, Allen N, Key TJ, Day NE, Linseisen J, Nagel G, Bergmann MM, Overvad K, Jensen MK, Tjonneland A, Olsen A, Bueno-de-Mesquita HB, Ocke M, Peeters PH, Numans ME, Clavel-Chapelon F, Boutron-Ruault MC, Trichopoulou A, Psaltopoulou T, Roukos D, Lund E, Hemon B, Kaaks R, Norat T, Riboli E (2006) Meat intake and risk of stomach and esophageal adenocarcinoma within the European Prospective Investigation Into Cancer and Nutrition (EPIC). J Natl Cancer Inst 98: 345–354 16507831 10.1093/jnci/djj071

[bib9] Goodman MT, Hankin JH, Wilkens LR, Lyu LC, McDuffie K, Liu LQ, Kolonel LN (1997) Diet, body size, physical activity, and the risk of endometrial cancer. Cancer Res 57: 5077–5085 9371506

[bib10] Gorbach SL, Goldin BR (1987) Diet and the excretion and enterohepatic cycling of estrogens. Prev Med 16: 525–531 3628202 10.1016/0091-7435(87)90067-3

[bib11] Guo WD, Chow WH, Zheng W, Li JY, Blot WJ (1994) Diet, serum markers and breast cancer mortality in China. Jpn J Cancer Res 85: 572–577 8063609 10.1111/j.1349-7006.1994.tb02398.x PMC5919531

[bib12] Holmes MD, Spiegelman D, Willett WC, Manson JE, Hunter DJ, Barbieri RL, Colditz GA, Hankinson SE (2000) Dietary fat intake and endogenous sex steroid hormone levels in postmenopausal women. J Clin Oncol 18: 3668–3676 11054440 10.1200/JCO.2000.18.21.3668

[bib13] Hou H, She Y, Ma Y, Hu C, Zheng M, Zhang S (1988) Investigations on methyl mercury contamination of fishes in the Second Songhua River. Biomed Environ Sci 1: 79–82 3268111

[bib14] Ip C (1997) Review of the effects of trans fatty acids, oleic acid, n-3 polyunsaturated fatty acids, and conjugated linoleic acid on mammary carcinogenesis in animals. Am J Clin Nutr 66: 1523s–1529s 9394710 10.1093/ajcn/66.6.1523S

[bib15] Jain MG, Rohan TE, Howe GR, Miller AB (2000) A cohort study of nutritional factors and endometrial cancer. Eur J Epidemiol 16: 899–905 11338120 10.1023/a:1011012621990

[bib16] Jiang QT, Le TKM, Chen K, Wong HL, Zheng JS, Giesy JP, Lo KKW, Yamashita N, Lam PKS (2005) Human health risk assessment of organochlorines associated with fish consumption in a coastal city in China. Environ Pollut 136: 155–165 15809117 10.1016/j.envpol.2004.09.028

[bib17] Kaaks R, Lukanova A, Kurzer MS (2002) Obesity, endogenous hormones, and endometrial cancer risk: a synthetic review. Cancer Epidemiol Biomarkers Prev 11: 1531–1543 12496040

[bib18] Koh WP, Yang HN, Yang HQ, Low SH, Seow A (2005) Potential sources of carcinogenic heterocyclic amines in the Chinese diet: results from a 24-h dietary recall study in Singapore. Eur J Clin Nutr 59: 16–23 15329677 10.1038/sj.ejcn.1602027

[bib19] La Vecchia C, Decarli A, Fasoli M, Gentile A (1986) Nutrition and diet in the etiology of endometrial cancer. Cancer 57: 1248–1253 3002600 10.1002/1097-0142(19860315)57:6<1248::aid-cncr2820570631>3.0.co;2-v

[bib20] Levi F, Franceschi S, Negri E, La Vecchia C (1993) Dietary factors and the risk of endometrial cancer. Cancer 71: 3575–3581 8490907 10.1002/1097-0142(19930601)71:11<3575::aid-cncr2820711119>3.0.co;2-0

[bib21] McCann SE, Freudenheim JL, Marshall JR, Brasure JR, Swanson MK, Graham S (2000) Diet in the epidemiology of endometrial cancer in Western New York (United States). Cancer Causes Control 11: 965–974 11142531 10.1023/a:1026551309873

[bib22] Nagao M, Ushijima T, Watanabe N, Okochi E, Ochiai M, Nakagama H, Sugimura T (2002) Studies on mammary carcinogenesis induced by a heterocyclic amine, 2-amino-1-methyl-6-phenylimidazo[4,5-*b*]pyridine, in mice and rats. Environ Mol Mutagen 39: 158–164 11921184 10.1002/em.10047

[bib23] Nair J, Vaca CE, Velic I, Mutanen M, Valsta LM, Bartsch H (1997) High dietary omega-6 polyunsaturated fatty acids drastically increase the formation of etheno-DNA base adducts in white blood cells of female subjects. Cancer Epidemiol Biomarkers Prev 6: 597–601 9264272

[bib24] Nakata H, Kawazoe M, Arizono K, Abe S, Kitano T, Shimada H, Li W, Ding X (2002) Organochlorine pesticides and polychlorinated biphenyl residues in foodstuffs and human tissues from china: status of contamination, historical trend, and human dietary exposure. Arch Environ Contamin Toxicol 43: 473–480 10.1007/s00244-002-1254-8 12399919

[bib25] Nowell S, Coles B, Sinha R, MacLeod S, Luke Ratnasinghe D, Stotts C, Kadlubar FF, Ambrosone CB, Lang NP (2002) Analysis of total meat intake and exposure to individual heterocyclic amines in a case-control study of colorectal cancer: contribution of metabolic variation to risk. Mutat Res 506-507: 175–185 12351157 10.1016/s0027-5107(02)00164-1

[bib26] Okochi E, Watanabe N, Shimada Y, Takahashi S, Wakazono K, Shirai T, Sugimura T, Nagao M, Ushijima T (1999) Preferential induction of guanine deletion at 5′-GGGA-3′ in rat mammary glands by 2-amino- 1-methyl-6-phenylimidazo[4,5-*b*]pyridine. Carcinogenesis 20: 1933–1938 10506107 10.1093/carcin/20.10.1933

[bib27] Olea N, Pazos P, Exposito J (1998) Inadvertent exposure to xenoestrogens. Eur J Cancer Prev 7: s17–s23 10866031 10.1097/00008469-199802001-00005

[bib28] Parkin DM (1989) Cancers of the breast, endometrium and ovary: geographic correlations. Eur J Cancer Clin Oncol 25: 1917–1925 2698807 10.1016/0277-5379(89)90373-8

[bib29] Parkin DM, Whelan SL, Ferlay J, Teppo L, Thomas DB (2003) Cancer Incidence in Five Continents Vol. VIII. Lyon, France: International Agency for Research on Cancer (IARC) press

[bib30] Petridou E, Kedikoglou S, Koukoulomatis P (2002) Diet in relation to endometrial cancer risk: a case–control study in Greece. Nutr Cancer 44: 16–22 12672637 10.1207/S15327914NC441_3

[bib31] Rojas E, Herrera LA, Poirier LA, Ostrosky-Wegman P (1999) Are metals dietary carcinogens? Mutat Res 443: 157–181 10415439 10.1016/s1383-5742(99)00018-6

[bib32] Rose DP, Connolly JM (1999) Omega-3 fatty acids as cancer chemopreventive agents. Pharmacol Ther 91: 414–428 10.1016/s0163-7258(99)00026-1 10576293

[bib33] Shields PG, Xu GX, Blot WJ, Fraumeni Jr JF, Trivers GE, Pellizzari ED, Qu YH, Gao YT, Harris CC (1995) Mutagens from heated Chinese and US cooking oils. J Natl Cancer Inst 87: 836–841 7791233 10.1093/jnci/87.11.836

[bib34] Shu XO, Yang G, Jin F, Kushi L, Liu D, Wen W, Gao YT, Zheng W (2004) Validity and reproducibility of food frequency questionnaire used in the Shanghai Women's Health Study. Eur J Clin Nutr 58: 17–23 14679362 10.1038/sj.ejcn.1601738

[bib35] Shu XO, Zheng W, Potischman N, Brinton LA, Hatch MC, Gao YT, Fraumeni Jr JF (1993) A population-based case–control study of dietary factors and endometrial cancer in Shanghai, People's Republic of China. Am J Epidemiol 137: 155–165 8452119 10.1093/oxfordjournals.aje.a116655

[bib36] Sinha R, Knize MG, Salmon CP, Brown ED, Rhodes D, Felton JS, Levander OA, Rothman N (1998) Heterocyclic amine content of pork products cooked by different methods and to varying degrees of doneness. Food Chem Toxicol 36: 289–297 9651045 10.1016/s0278-6915(97)00159-2

[bib37] Sinha R, Peters U, Cross AJ, Kulldorff M, Weissfeld JL, Pinsky PF, Rothman N, Hayes RB (2005) Meat, meat cooking methods and preservation, and risk for colorectal adenoma. Cancer Res 65: 8034–8041 16140978 10.1158/0008-5472.CAN-04-3429

[bib38] Sinha R, Rothman N, Salmon CP, Knize MG, Brown ED, Swanson CA, Rhodes D, Rossi S, Felton JS, Levander OA (1998) Heterocyclic amine content in beef cooked by different methods to varying degrees of doneness and gravy made from meat drippings. Food Chem Toxicol 36: 279–287 9651044 10.1016/s0278-6915(97)00162-2

[bib39] Spingarn NE, Kasai H, Vuolo LL, Nishimura S, Yamaizumi Z, Sugimura T, Matsushima T, Weisburger JH (1980) Formation of mutagens in cooked foods. III. Isolation of a potent mutagen from beef. Cancer Lett 9: 177–183 7226149 10.1016/0304-3835(80)90084-1

[bib40] Sturgeon SR, Brock JW, Potischman N, Needham LL, Rothman N, Brinton LA, Hoover RN (1998) Serum concentrations of organochlorine compounds and endometrial cancer risk (United States). Cancer Causes Control 9: 417–424 9794174 10.1023/a:1008823802393

[bib41] Terry PD, Rohan TE, Wolk A (2003) Intakes of fish and marine fatty acids and the risk of cancers of the breast and prostate and of other hormone-related cancers: a review of the epidemiologic evidence. Am J Clin Nutr 77: 532–543 12600840 10.1093/ajcn/77.3.532

[bib42] Terry P, Vainio H, Wolk A, Weiderpass E (2002) Dietary factors in relation to endometrial cancer: a nationwide case-control study in Sweden. Nutr cancer 42: 25–32 12235647 10.1207/S15327914NC421_4

[bib43] Terry P, Wolk A, Vainio H, Weiderpass E (2002) Fatty fish consumption lowers the risk of endometrial cancer – a nationwide case–control study in Sweden. Cancer Epidemiol Biomarkers Prev 11: 143–145 11815413

[bib44] van Vliet T, Katan MB (1990) Lower ratio of n-3 to n-6 fatty acids in cultured than in wild fish. Am J Clin Nutr 51: 1–2 2296923 10.1093/ajcn/51.1.1

[bib45] Wakabayashi K, Nagao M, Esumi H, Sugimura T (1992) Food-derived mutagens and carcinogens. Cancer Res 52(suppl): 2092s–2098s 1544146

[bib46] Weiderpass E, Adami HO, Baron JA, Wicklund-Glynn A, Aune M, Atuma S, Persson I (2000) Organochlorines and endometrial cancer risk. Cancer Epidemiol Biomarker Prev 9: 487–493 10815693

[bib47] Willett WC (1998) Nutritional Epidemiology: Monographs in Epidemiology and Biostatistics, pp 148–156. New York: Oxford University Press

[bib48] Wong KY, Su J, Knize MG, Koh WP, Seow A (2005) Dietary exposure to heterocyclic amines in a Chinese population. Nutr Cancer 52: 147–155 16201846 10.1207/s15327914nc5202_5

[bib49] Zheng W, Kushi LH, Potter JD, Sellers TA, Doyle TJ, Bostick RM, Folsom AR (1995) Dietary intake of energy and animal foods and EC incidence. The Iowa women's health study. Am J Epidemiol 142: 388–394 7625403 10.1093/oxfordjournals.aje.a117646

[bib50] Zheng W, Gustafson DR, Sinha R, Cerhan JR, Moore D, Hong CP, Anderson KE, Kushi LH, Sellers TA, Folsom AR (1998) Well-done meat intake and the risk of breast cancer. J Natl Cancer Inst 90: 1724–1729 9827527 10.1093/jnci/90.22.1724

[bib51] Zimmerli B, Rhyn P, Zoller O, Schlatter J (2001) Occurrence of heterocyclic aromatic amines in the Swiss diet: analytical method, exposure estimation and risk assessment. Food Addit Contam 18: 533–551 11407752 10.1080/02652030119545

